# Illinois State Medical Society, and the National Association

**Published:** 1860-04

**Authors:** 


The Illinois State Medical Society and the National Association.—At the last meeting of the Illinois State Medical Society a movement was made, looking to *reform* in the manner of electing the President of the National Association. A resolution relating to this matter was introduced, as was understood, at the instance of Dr. N. S. Davis, but did not pass. At present the Association has the right of choosing its officers from any part of the Union, although for obvious and proper reasons the Presidents have most frequently been taken from the State in which the meeting was held. Dr. Davis proposes to make a rule prohibiting such choice, and disfranchising the members of the profession in the particular State or city in which the meeting may be held, and urges his friends to rally and put it through.

This is an ingenious move. In the first place it might make his chances of election better at New Haven than they were at Washington and at Louisville, where he did not succeed. Secondly, if the Association should ever meet in this State it would prevent the election of any other person. If this resolution passes, the next move may be to invite the Association to Chicago, otherwise not.

It is melancholy to see so much attention paid to an unimportant matter, to the neglect of the scientific interest of the State and National Societies. But it is in accordance with the peculiar system of elevating the profession advocated in a certain set just at present.

For ourselves, we cannot but express a hope that our friends will, whenever consistent with their interests, attend these meetings, and that they will take pains to arrange, for the purpose of being communicated, any valuable facts or observations which may have occurred to them during the year. Thus interest may be added to the meetings, and value to the transactions.

---

☞ The annual meeting of the Illinois State Medical Society will take place on the third Tuesday of May, 1860.

