# Correction—Meeting of Illinois State Medical Society

**Published:** 1860-05

**Authors:** 


☞ Correction.—By an oversight of the compositor, in our April number, what was designed to be a notice of the meeting of the Indiana State Medical Society, was made to read, the "Illinois State Medical Society." The Indiana Society meets on the third Tuesday in May, 1860, at Indianapolis; the delegates from the Illinois Society being Drs. Brainard and Miller, of Chicago, and Dr. H. W. Davis, of Paris.

The Illinois State Society meets at Paris, Edgar Co., on the second Tuesday in May.—8th.

